# Potential *in vitro* toxicity of fusel oil components

**DOI:** 10.3389/ftox.2026.1979030

**Published:** 2026-10-13

**Authors:** Junwei Zhao, Zezhi Li, Xiaoxia Xia, Xiang Li

**Affiliations:** Beijing Life Science Academy, Beijing, China

**Keywords:** cells, cytotoxicity, fusel oil, *in vitro*, toxicity assessment

## Abstract

Fusel oil is a by-product during the fermentation of alcoholic beverage, mainly including n-propanol, isobutanol, isopentanol, and others. Long-term or excessive intake of them may cause potential harm to human health. In the present work, we preliminarily evaluated the cytotoxicity of n-propanol and isobutanol, main components of fusel oil, in human fetal astrocytes (SVGp12), human microvascular endothelial cells (HMEC-1), and human microglial cells (HMC3). The results showed that both n-propanol and isobutanol exhibited cytotoxicity in all three types of cells, and the release of proinflammatory cytokines (IL-6 and IL-8) significantly increased in the cells exposed to fusel oil components. The transepithelial electrical resistance (TEER) values were decreased and the permeability was increased in HMEC-1 cells exposed to fusel oil components. These results indicate that fusel oil components may cause potential harm to human health.

## 1 Introduction

Fusel oil affects the quality and safety of alcoholic beverage. The components of fusel oil in alcoholic beverage usually include n-propanol, n-butanol, isobutanol, and isopentanol, and others (Chen and David, 1974; Neale, 1988). These substances are produced by microbial metabolism during the fermentation process of alcoholic beverage (Walther and Francois, 2016). They can enhance the aroma and taste of alcoholic beverage at certain concentrations. For example, 1-propanol and 1-butanol are as aroma compounds in the liquor, both of which were in the 100 mg/L range. The concentration of 1-propanol in chixiang aroma-type liquor is 88.2 mg/L (Fan et al., 2015). In the second-class liquor, the concentrations of 1-propanol and 1-butanol are 329.8 mg/L and 157.8 mg/L respectively (Lin et al., 2024). Some components could be detected in the human blood after drinking alcoholic beverage (Barz et al., 1990; Schuberth, 1991). Ethanol, methanol, 2-propanol, and acetone were detected in clinical and forensic evaluation of alcohol intoxication, and measured alcohol levels ranged 1.2–597 mg per 100 mL in blood samples of the patients (Bulut et al., 2026). Their potential hazards to human health cannot be ignored. High-quality alcoholic beverages exhibit a low propensity for inducing adverse physiological effects. The consumption of impure or low-quality alcoholic beverages, which contain elevated levels of fusel oil, can potentially induce adverse effects on the central nervous system of the body when consumed in excess. The symptoms of adverse effects include dizziness, headache, drowsiness, uncoordinated movements, memory loss, and decreased attention (Stobbe, 1981). This daily life phenomenon indicates that fusel oil components might have potential adverse effects on the nervous system. However, there exists a significant scarcity of research efforts addressing the toxic effects of fusel oil.

With the promotion of the 3R principle (Replacement, Reduction, Refinement) for laboratory animals, *in vitro* models are the preferred choice for the rapid screening and evaluation of compound toxicity. In the present work, we preliminarily evaluated the *in vitro* toxicity of n-propanol and isobutanol, main components of fusel oil, in human fetal astrocytes (SVGp12), human microvascular endothelial cells (HMEC-1), and human microglial cells (HMC3). In this study, we selected n-propanol and isobutanol, the major constituents of fusel oil, as representative compounds for preliminary *in vitro* toxicity assessment. Due to the difficulty to directly determine the actual exposure level of humans to fusel oil components, the setting of the exposure concentrations for the two components refers to the alcohol concentration levels in the blood after drinking. The alcohol concentration in human blood after drinking is typically measured at a level of mg per 100 mL range. The standard for drink-driving is generally defined as an alcohol content of more than 20 mg per 100 mL of human blood, and in some countries the limit is 80 mg per 100 mL (Issa and Khattab, 2024). In the current toxicity testing, the exposure concentration range of the two components was within the 0–5 mg/mL range, which is consistent with the alcohol concentrations previously reported by Bulut et al. (Bulut et al., 2026) in their analysis of patients with alcohol intoxication.

## 2 Materials and methods

### 2.1 Materials

N-propanol and isobutanol were purchased from Shanghai Aladdin Biochemical Technology Co., Ltd. (China). SVGp12, HMEC-1 and HMC3 cells were purchased from the Cell Bank of the Chinese Academy of Sciences (China). DMEM medium, MCBD131 medium, fetal bovine serum, N-2 Supplement, B-27 supplement, HEPES, GlutaMAX I supplement, hEGF and penicillin-streptomycin were purchased from Thermo Fisher Scientific (UK). FITC-dextran (molecular weight: 40 kDa) was obtained from MedChemExpress (USA). Cell counting Kit-8 were purchased from Macklin (China). Human IL-6 and IL-8 ELISA Kits were purchased from Boster Biological Technology Co., Ltd. (China). 96-well plates and 12-well transwell plates were obtained from Corning (UK). Microplate reader was purchased from Thermo Fisher Scientific (UK). Electrical resistance meter EVOM3 were purchased from World Precision Instruments (USA).

### 2.2 Cytotoxicity assay

After thawing, cells were passaged three times, and those in the logarithmic growth phase were used for subsequent experiments. The cell suspensions of SVGp12, HMEC-1 and HMC3 were respectively seeded into 96-well plates at 100 μL per well, with a cell concentration of 1.0 × 10^5^/mL. After seeding, the plates were placed in an incubator (37 °C, 5% CO_2_) for overnight culture. The initial 5 mg/mL n-propanol solution and isobutanol solution were subjected to two-fold serial dilution to generate eight concentrations (0, 0.078125, 0.15625, 0.3125, 0.625, 1.25, 2.5, and 5 mg/mL). The three types of cells were respectively exposed to the eight concentrations of n-propanol or isobutanol for 24 h. Then, 10 μL of CCK-8 solution was added to each well of the plate, and the plates were protected from light and incubated at 37 °C for 1 h. The optical density was measured at 450 nm using microplate reader. The half maximal inhibitory concentration (IC_50_) was calculated by using Graphpad Prism 9.5.0 (USA).

### 2.3 Enzyme lined immunosorbent assay (ELISA)

SVGp12 cells were exposed to n-propanol or isobutanol at concentration of 0.078125 mg/mL, HMEC-1 cells were treated with n-propanol or isobutanol at 0.3125 mg/mL, and HMC3 cells were incubated with n-propanol or isobutanol at 0.15625 mg/mL, with all treatments lasting for 24 h. The control group was administrated without the addition of n-propanol or isobutanol. The cell culture supernatant was harvested and subjected to centrifugation at 4 °C to pellet cellular debris. The resulting clear supernatant was then collected for subsequent analysis.The levels of IL-6 and IL-8 in the samples were determined with human-specific ELISA kits. The experimental procedures were carried out in accordance with the instruction of ELISA Kits.

### 2.4 Transepithelial electrical resistance (TEER) measurement

In the TEER experiment, HMEC-1 cells were treated with 0.3125 mg/mL of n-propanol or isobutanol for 24 h, respectively. The cell suspensions of HMEC-1 were seeded into 12-well transwell plate at 1 mL per insert, with a cell concentration of 1.0 × 10^5^/mL, and HMEC-1 cells were incubated for 24 h in an incubator (37 °C, 5% CO_2_). The TEER values were measured at 0 h, 1 h, 2 h, 3 h, 4 h, 5 h, 6 h and 24 h respectively by using the transcutaneous electrical resistance meter.

Prior to TEER measurement, inserts were equilibrated at 37 °C for 30 min. The electrodes were inserted vertically into the transwell insert, with the long electrode suspended in the lower chamber and the short electrode positioned above the membrane in the upper chamber, ensuring that the coated tips of both electrodes were fully immersed in the culture medium. Readings were taken after the values stabilized, and all measurements were subtracted from the blank control values. Cell-free blank transwell inserts were set up in parallel. The membrane resistance was corrected using the formula: TEER = (total resistance − blank resistance) × effective membrane area (1.12 cm^2^).

### 2.5 Permeability evaluation

In the permeability assay, HMEC-1 cells were exposed to 0.3125 mg/mL of n-propanol or isobutanol for 24 h, respectively. The cell suspensions of HMEC-1 with a cell concentration of 1.0 × 10^5^/mL were added into the inserts of 12-well transwell plate, and HMEC-1 cells were incubated for 24 h in an incubator (37 °C, 5% CO_2_). Then, the cells were exposed to n-propanol solution or isobutanol at a concentration causing a cell survival rate of approximately 80% for 24 h. Adding the solution of FITC-dextran (MW 40000) into the inserts, and samples were collected at 15, 30, 45, 60, 90, and 120 min respectively. Then the fluorescence values of the samples were detected using a microplate reader and calculated the apparent permeability coefficient (*P*_app_).

The upper and lower chambers were gently washed twice with pre-warmed HBSS-HEPES buffer. Subsequently, 1.5 mL of HBSS-HEPES buffer was added to the lower chamber, and the plate was equilibrated in an incubator for 15–30 min. FITC-dextran (4 kDa) was applied to the upper chamber at a final concentration of 1 mg/mL (dissolved in HBSS-HEPES buffer). Samples of 300 μL were collected from the lower chamber at 0, 15, 30, 45, 60, 90, and 120 min, with an equal volume of fresh buffer being replaced after each sampling. The apparent *P*app was calculated using the standard formula: *P*app (cm/s) = (dQ/dt)/(A × C_0_), where dQ/dt is the slope of the cumulative permeated amount versus time curve (μg/s), A is the effective surface area of the transwell membrane (cm^2^), and C_0_ is the initial concentration in the upper chamber (μg/mL).

### 2.6 Statistical analysis

The data were expressed as mean ± SD. The differences in the release levels of proinflammatory cytokines (IL-6 and IL-8) in the cells with or without fusel oil components exposure were analyzed by a paired t-test. For *P*app, statistical analyses were performed using one-way analysis of variance (ANOVA), followed by Dunnett’s *t*-test for *post hoc* pairwise comparisons. The IC_50_ values were determined by fitting the dose-response curves via nonlinear regression. *P* < 0.05 was considered as statistically significant.

## 3 Results

### 3.1 Cytotoxicity of fusel oil components to the cells

The results showed that both n-propanol and isobutanol exhibited cytotoxicity in all three types of cells, and there was a clear dose-effect relationship (Figure 1). The IC_50_ values of n-propanol for SVGp12, HMEC-1 and HMC3 cells are 0.49 ± 0.18 mg/mL, 0.99 ± 0.05 mg/mL and 1.82 ± 0.23 mg/mL respectively. The IC_50_ values of isobutanol for SVGp12, HMEC-1 and HMC3 cells are 0.33 ± 0.08 mg/mL, 1.01 ± 0.06 mg/mL and 2.04 ± 0.45 mg/mL respectively. SVGp12 cells were more sensitive to the toxicity of fusel oil components.

**FIGURE 1:**
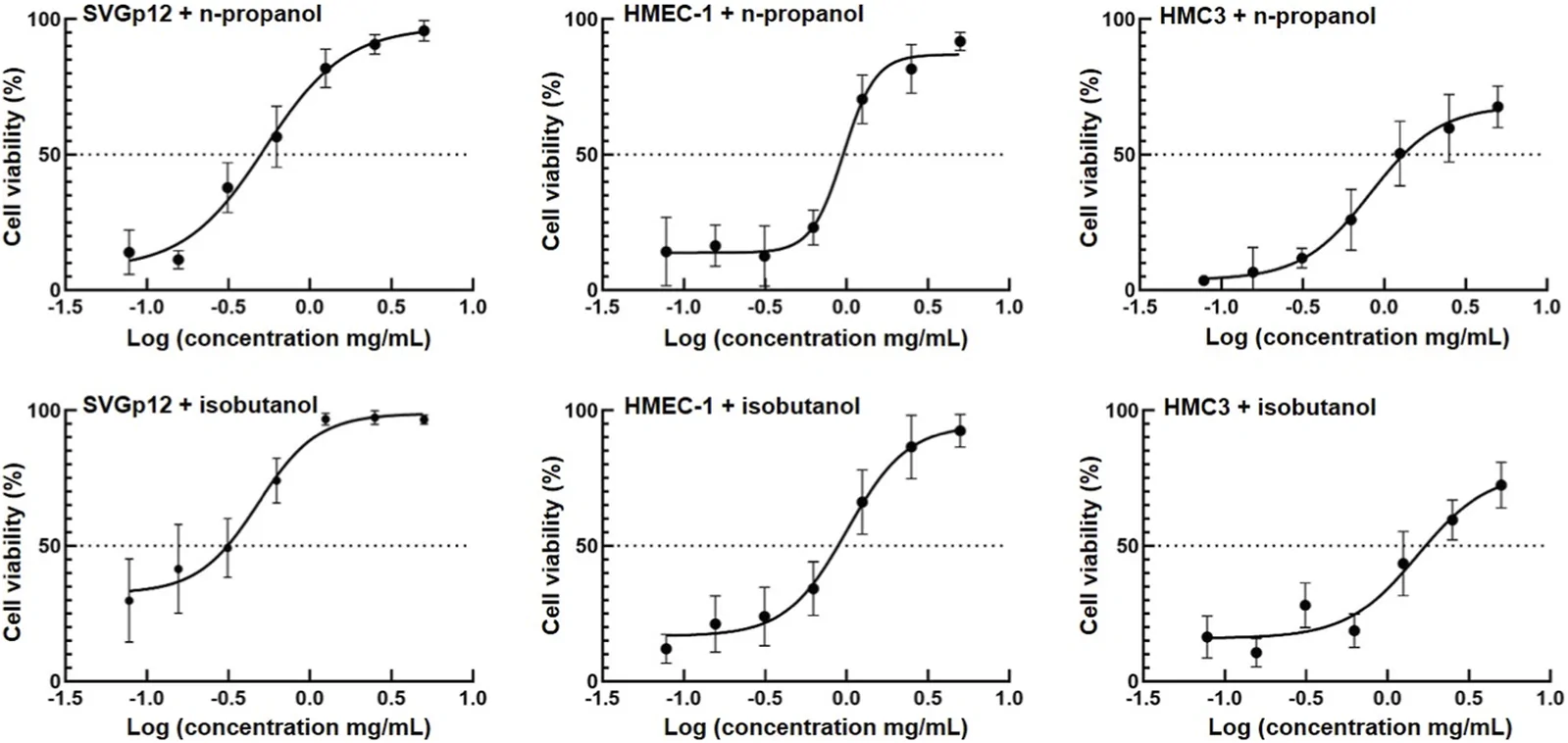
The cytotoxicity of n-propanol and isobutanol in three types of cells, SVGp12, HMEC-1 and HMC3. Each component was exposed to each type of cells for three independent experiments. And each experiment used one 96-well plate, with five repetitional wells for each concentration in each plate. The data were expressed as mean ± SD.

### 3.2 The release of proinflammatory cytokines in the cells exposed to fusel oil components

The results of ELISA indicated that the release of proinflammatory cytokines (IL-6 and IL-8) significantly increased in all three types of cells exposed to n-propanol or isobutanol (Figure 2).

**FIGURE 2:**
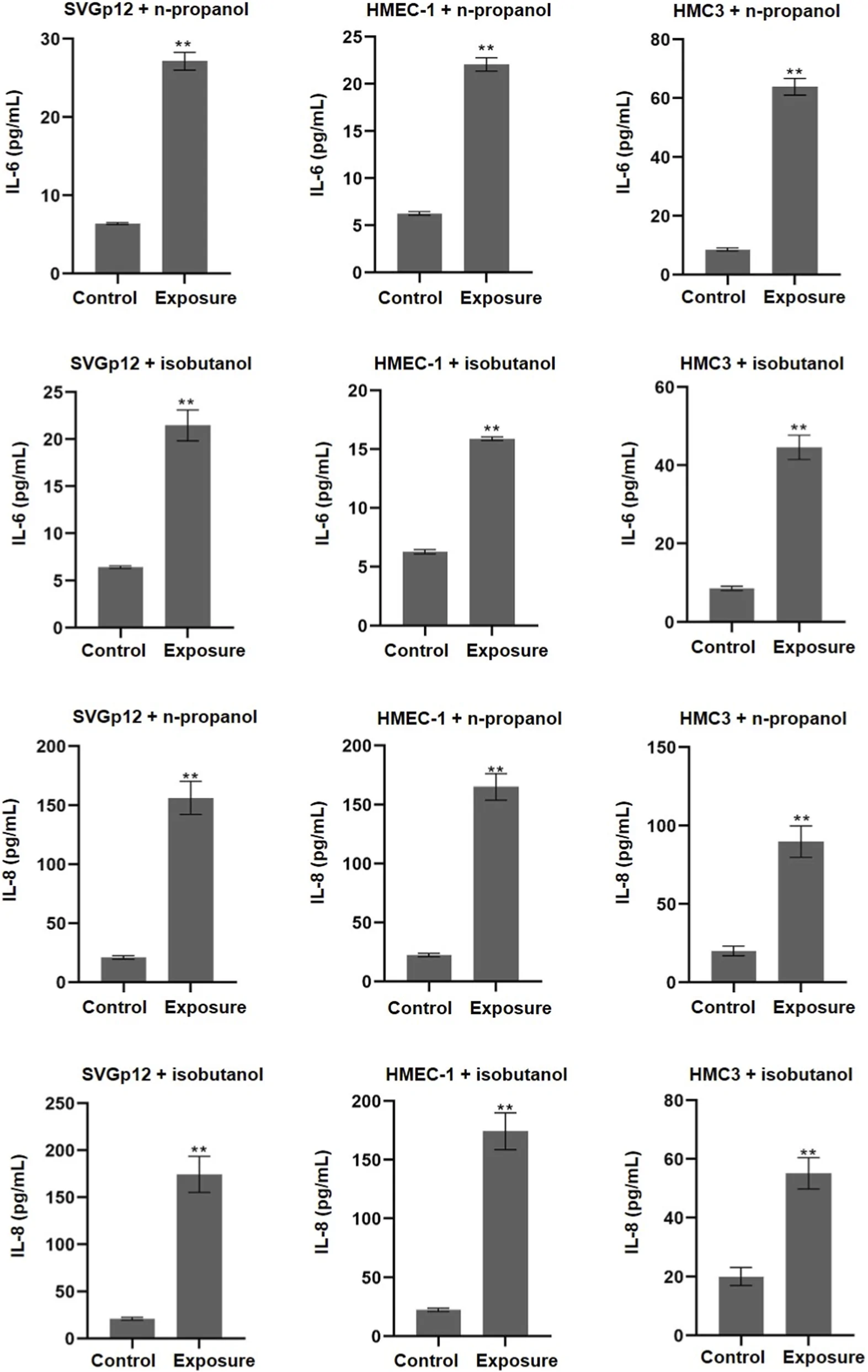
The release levels of IL-6 and IL-8 in three types of cells, SVGp12, HMEC-1 and HMC3 with n-propanol or isobutanol exposure. Each assay was conducted on three independent experiments, and the data were expressed as mean ± SD. **P < 0.01.

### 3.3 TEER measurement and permeability evaluation

The blood-brain barrier (BBB) plays a crucial biological role in maintaining the normal physiological state of the central nervous system. The BBB is mainly composed of vascular endothelial cells. Integrity, permeability and barrier function are the fundamental physiological functions of the BBB. HMEC-1 cells cultured with transwells were as *in vitro* models for TEER measurement and permeability evaluation after fusel oil components exposure. Figure 3A showed that the TEER values of the cells began to decrease after exposure to fusel oil components, and the TEER values continued to decrease throughout the 24-h period of exposure. Figure 3B showed that fusel oil components induced an increase in the apparent *P*_app_.

**FIGURE 3:**
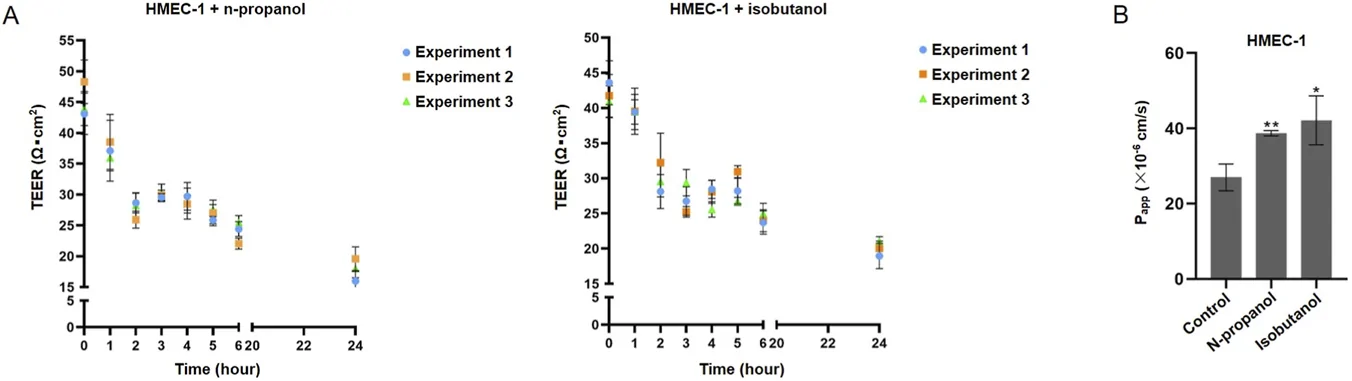
Effects of fusel oil components on the TEER and the permeability of HMEC-1 cells. **(A)** The TEER values of HMEC-1 cells exposed to fusel oil components. Each assay was conducted on three independent experiments, and four measurements were conducted in each experiment. The data were expressed as mean ± SD. **(B)** The permeability of HMEC-1 cells exposed to fusel oil components. Each assay was conducted on three independent experiments. The data were expressed as mean ± SD. Compared to control: *P < 0.05, **P < 0.01.

## 4 Discussion

Previous studies found that n-propanol and n-butanol had acute toxic effects on mouse neuroblastoma cells, affecting the release of neurotransmitters and causing changes in cell excitability (Walum and Peterson, 1983). In the sub-chronic isobutanol exposure experiment, no specific, persistent or progressive effects on the nervous system of SD rats were observed, while the response of experimental animals to external stimuli slightly decreased during acute exposure (Li et al., 1999). N-butanol could cause toxic effects on multiple organs of experimental animals via enteral and inhalant administration (Sinitsyna et al., 2003), and could inhibit the proliferation of astrocytes in fetal rat brains, having potential developmental neurotoxic effects (Bale and Lee, 2016). Isopropanol showed potential reproductive and developmental toxicity in rats through oral gavage or drinking water administration (Faber et al., 2008). N-butanol and isobutanol acted on different targets in the brain (Bale and Lee, 2016). There is a report that the fusel oil was suppressed taste-aversion behavior in animal hangover models (Hori et al., 2003). However, there were studies indicating that exposure to n-butanol had no significant impacts on neural development of rats (Nelson et al., 1989; Ema et al., 2005). These components might also further exacerbate neuronal damage by changing the permeability of cerebral blood vessels, inhibiting the activity of antioxidant enzymes, and inducing oxidative stress responses (Yamada et al., 2015). While limited studies have suggested the potential toxic effects of individual component of fusel oil on the nervous system, the findings have been inconsistent, and the overall evidence supporting neurotoxicity remains limited. The results of toxicity assessment of fusel oil components reported in some literature are shown in Table 1. In addition, there is a lack of research on dose effects and the impact of fusel oil components on the function of the brain (Segal et al., 2020).

**TABLE 1:** Examples of toxicity assessment of fusel oil components.

Component	Test subjects	Toxic effects	References
n-butanol	Chick embryo	Increased corneal opacity, nerve damage	(Mclaughlin et al., 1964)
n-butanol, n-propanol	Mouse neuroblastoma cells (C1300)	Acute toxic effects, release of neurotransmitters and changes in cell excitability	(Walum and Peterson, 1983)
n-butanol	Rats	Foetotoxic effect	(Sitarek et al., 1994)
isobutanol	Sprague-Dawley rats	No morphological or behavioral effects, a slight decrease in response to external stimuli	(Li et al., 1999)
n-butanol	Albino rats	Membrano-, hepato-, adrenotoxic, neurotoxic effects	(Sinitsyna et al., 2003)
1-butanol	Rats	Developmental toxic only at maternal toxic doses	(Ema et al., 2005)
n-propanol, isobutanol	Human cell lines: SVGp12 (astrocytes), HMEC-1 (endothelial), and HMC3 (microglia)	Cytotoxicity, proinflammatory, the toxic effects on endothelial barrier integrity	This study

In the present assays, the results indicated that n-propanol and isobutanol had cytotoxicity in endothelial cells, astrocytes and microglial cells. The release of proinflammatory cytokines (IL-6 and IL-8) significantly increased in the cells exposed to fusel oil components. The 3 cell lines used represents the constituent cells of the neurovascular unit of the central nervous system. Subsequent evaluation was conducted on the impact of fusel oil components on the barrier function of vascular endothelial cells. Both n-propanol and isobutanol induced a decrease in the TEER and an increase in the permeability of HMEC-1 cells. These results suggest that fusel oil components may have an adverse effect on the BBB *in vivo*. Further research is required to identify specifically which brain structures and types of neural cells are most affected by fusel oil. The selection of exposure doses for fusel oil components is one of the limitations of this study. The dosages used in the *in vitro* experiments differs significantly from the actual intake after human drinking. In a forensic chemistry analyses reported, the blood alcohol level was detected within the range of 0.12%–0.31% (1.2–3.1 mg/mL) in the cases of death attributed to alcohol/alcohol intoxication (Puittinen et al., 2024). Although the testing concentrations range of 0–5 mg/mL was based on the blood alcohol concentrations reported for the patients with alcohol intoxication (Bulut et al., 2026), it does not represent the actual exposure levels of these two components. In the cytotoxicity assay, the higher concentrations of n-propanol and isobutanol were selected to determine the IC_50_ values. The extrapolation of the *in vitro* data to the human studies also requires special attention.

Based on the present results, the fusel oil components present in alcoholic beverage should be given attention and they might cause potential harm to human health. These results could provide theoretical basis for the hazard identification of potential harmful substances in alcoholic beverage as well as the limit control of fusel oil components. Reducing the content of fusel oil might improve the quality of alcoholic beverage and alleviate the discomfort after drinking.

Despite being a preliminary investigation, the current study has several limitations. First, It should be noted that HMEC-1 is not a brain-derived endothelial cell line; while it serves as a useful preliminary endothelial model, it does not fully recapitulate the complexity of the blood–brain barrier. Therefore, its use as a surrogate for the BBB endothelium lacks tissue specificity. In assessing the effects on the BBB, consideration should also be given to other cell types constituting the BBB. Second, A considerable gap exists between the concentrations used in *in vitro* experiments and those occurring *in vivo* following real-world exposure, which constitutes a well-recognized limitation of cellular-level investigations. The actual concentrations reaching human tissues are governed by the integrated effects of absorption, distribution, first-pass metabolism, and clearance rates. Therefore, the present findings should be interpreted as preliminary evidence for the potential physiological actions of fusel oils at the cellular level, and caution should be exercised in extrapolating these results to human safe exposure limits or clinical implications. The present findings demonstrate potential cytotoxic, inflammatory, and barrier-disrupting effects *in vitro*, but they cannot by themselves establish human health risks or directly predict effects following consumption of alcoholic beverages. Third, It should be acknowledged that the effects of n-butanol and isopentanol, two key constituents of fusel oil, on endothelial barrier function were not examined in the present study. Future investigations will incorporate these compounds to enable a more comprehensive toxicological evaluation of fusel oil components. Fourth, The experiments were performed with three independent replicates (n = 3), which confers limited statistical power. Consequently, the findings should be considered preliminary and will require validation through future studies with a larger sample size. Fifth, The CCK-8 assay employed in this study indirectly reflects cellular metabolic activity by measuring mitochondrial dehydrogenase activity, serving as a functional endpoint rather than a direct quantitative measure of cell death. The inherent limitation of this method lies in the fact that a decrease in metabolic activity may result from either inhibition of cell proliferation or cell death, making it difficult to definitively distinguish between these two processes.

To further strengthen the reliability and tissue specificity of our conclusions, subsequent experiments will employ a human brain-derived microvascular endothelial cell line (e.g., hCMEC/D3) to validate the key findings of this study. In future research, establishing a scientific and reasonable evaluation model designed to mimic the microenvironment within the brain, and exploring the neurotoxic effects along with their mechanisms of action for fusel oil components, can provide experimental evidence and technical support for identifying chemical hazards and conducing risk assessment of food-derived toxicants. Future research could explore oxidative stress, NF-κB signaling, apoptosis, and tight-junction alterations as potential areas for in-depth mechanism studies. BBB plays a crucial biological role in maintaining the normal physiological state of the central nervous system. *In vitro* BBB microfluidic model can be regarded as a candidate model. When designing and constructing the BBB model, some aspects, are not limited to these listed here, that may need to be considered: 1) Co-culture of various cells such as vascular endothelial cells, astrocytes and pericytes, which can mimic the cellular structure of the BBB *in vivo*. In the context of replicating realistic cellular environments of the BBB within a microfluidic chip, incorporating multiple cell types can enhance the structural mimicry of natural tissues, providing more accurate and applicable experimental results. However, this increased complexity poses challenges in constructing and maintaining a BBB chip. 2) Biomimicry of the integrity, permeability and function of the BBB. Integrity, permeability and barrier function are the three fundamental physiological functions of the BBB. The complete replication of BBB physiological functions is crucial for obtaining reliable experimental results. 3) With a concentration gradient generation unit for fusel oil components exposure. This unit enables simultaneous exposure of different doses of components on the chip, which is necessary for obtaining the dose-response data of toxicity endpoints. It also increases the testing throughput of the chip and facilitates the comparability of results under the same experimental conditions. 4) Continuous supply of cell culture medium and online collection of cellular metabolites. This ensures the growth and maintenance of cells on the chip, while simultaneously collecting metabolites online for the analysis of biomarkers at different time points. That is, under a single exposure experiment, biological samples can be obtained during continuous exposure period, which is very important for studying time-dependent data of toxicity endpoints as well as the experimental efficiency improvement. Furthermore, the integration of *in situ* detection methods, such as immunofluorescence and real-time permeability monitoring, alongside multi-omics technologies, with the BBB chip exposure enables comprehensive analysis of toxicity endpoints and underlying toxicological mechanisms. In addition, the BBB chip can be used to evaluate the toxic effects of fusel oil. However, the challenges in practical application research will involve how to separate and quantify fusel oil components from alcoholic beverage, as well as the selection of an appropriate microfluidic panel for this purpose. With *in vitro* BBB chip as the core, combined with fusel oil separation chip (if possible), this system will have the potential to offer an optional platform for the risk assessment of fusel oil components in alcoholic beverage.

## Data Availability

The original contributions presented in the study are included in the article/supplementary material, further inquiries can be directed to the corresponding authors.
